# The Juvenile Hafnium Isotope Signal as a Record of Supercontinent Cycles

**DOI:** 10.1038/srep38503

**Published:** 2016-12-07

**Authors:** Nicholas J. Gardiner, Christopher L. Kirkland, Martin J. Van Kranendonk

**Affiliations:** 1 Centre for Exploration Targeting–Curtin Node, Department of Applied Geology, Western Australian School of Mines, Curtin University, Perth, WA 6102, Australia; 2 Australian Research Council Centre of Excellence for Core to Crust Fluid Systems, Australia; 3 School of Biological, Earth and Environmental Sciences, University of New South Wales Australia, Kensington, NSW 2052, Australia

## Abstract

Hf isotope ratios measured in igneous zircon are controlled by magmatic source, which may be linked to tectonic setting. Over the 200–500 Myr periodicity of the supercontinent cycle - the principal geological phenomenon controlling prevailing global tectonic style - juvenile Hf signals, i.e. most radiogenic, are typically measured in zircon from granites formed in arc settings (crustal growth), and evolved zircon Hf signals in granites formed in continent-collision settings (crustal reworking). Interrogations of Hf datasets for excursions related to Earth events commonly use the median value, however this may be equivocal due to magma mixing. The most juvenile part of the Hf signal is less influenced by crustal in-mixing, and arguably a more sensitive archive of Earth’s geodynamic state. We analyze the global Hf dataset for this juvenile signal, statistically correlating supercontinent amalgamation intervals with evolved Hf episodes, and breakup leading to re-assembly with juvenile Hf episodes. The juvenile Hf signal is more sensitive to Pangaea and Rodinia assembly, its amplitude increasing with successive cycles to a maximum with Gondwana assembly which may reflect enhanced subduction-erosion. We demonstrate that the juvenile Hf signal carries important information on prevailing global magmatic style, and thus tectonic processes.

Over Earth history, the Hafnium (Hf) isotopic ratio (^176^Hf/^177^Hf), as measured in the refractory mineral zircon, reveals a strong genetic connection to the dominant global tectonic regime^1^. Zircon is ubiquitously carried in granitic magmas; its Hf isotope signal is strongly controlled by magmatic source, itself linked to tectonic setting. Thus, secular excursions in zircon Hf isotopic data relative to depleted mantle (DM) have been linked to tectonic processes that give rise to particular types of magmatism^1^,^2^. Over global averages, evolved Hf signatures, i.e. those with negative εHf values, are typically measured within S-type granites that have their genesis in continental collision settings, whereas juvenile Hf signatures showing positive εHf values – i.e. radiogenic signals close to that of DM - are typically measured in I-type granites that largely form in arc-type settings^3^,^4^.

Critical interrogation of global zircon Hf isotopic datasets has addressed questions on: the rate of crust formation, and the onset of modern-style plate tectonics^3^,^5^,^6^,^7^,^8^; the balance of crust formation versus destruction during supercontinent assembly^4^,^9^; and inter-supercontinent comparisons^10^,^11^,^12^,^13^,^14^. Such evaluation has generally focused on the balance between juvenile and evolved Hf signals through time, which has been accomplished through documenting the secular excursion in the median Hf isotopic value^4^, or interrogation of Hf evolutionary arrays through time to extract information on the timing of new crust generation^6^,^8^. However, deciphering the source of evolved Hf ratios can be challenging due to magma mixing and hybrid sources^15^. The most juvenile (i.e. most radiogenic) part of the global Hf dataset records a signature from crust that has recently been involved in a fractionation event from a DM reservoir, and may therefore record the formation of new crust. The most juvenile signal potentially represents the least mixed part of the Hf record, and it may therefore be a more accurate archive of Earth’s predominant magmatism - and by extension of prevailing tectonic style - than the median signal. Hence, we address the question: does the juvenile part of the global Hf record preserve greater fidelity to secular changes in tectonic cycles than the median? In addition, can this juvenile Hf component be used to refine our understanding of global tectonic cycles?

In order to address these questions, we constructed timeseries representing two different juvenile signals (99% and 95%), and the median signal (50%), of the global Hf record. We assessed their sensitivities to Earth evolution by statistically linking the variability of these signals to the supercontinent cycle over the last 2.2 Ga. The difference between the median and the juvenile timeseries reflects the spread of Hf values at a given point during Earth’s history. Through comparison of how the different signals track the assembly of four successive supercontinents, we demonstrate that the juvenile part of the Hf record is more sensitive to the assembly of both the supercontinents Pangaea and Rodinia than the median. We propose that the juvenile Hf signal represents a more sensitive way to interrogate global Hf datasets, with respect to magmatic style, than the median signal, and which may be conceptually linked to prevailing tectonic processes.

## Hf Isotopes: A Background

Newly-generated crust extracted from the mantle may have an initial ^176^Hf/^177^Hf ratio modelled as that of depleted mantle (DM), typically expressed as evolution from CHUR at 4.5 Ga to an initial ^176^Hf/^177^Hf similar to that of average mid ocean ridge basalt^16^. A juvenile zircon Hf signal is measured in magmas derived from material recently extracted from DM with minimal to no older crustal contamination^6^. Hf analyses recording a lower initial ^176^Hf/^177^Hf than DM may reflect a source of reworked existing older crust with no significant new mantle input, or alternatively a mix between juvenile and evolved sources. Although an array along an evolution line defined by a typical crustal source ^176^Lu/^177^Hf ratio may be considered as evidence of reworking a single source^17^, it may still reflect cryptic mixing or early homogenization processes, i.e. an earlier mixing event^15^. Independent constraints such as oxygen isotopes may be used to screen Hf data for a single source^3^,^6^, although such screening can still be ineffective^18^. Hence, the zircon Hf isotopic system is a sensitive monitor of the degree to which a melt source reflects juvenile mantle addition versus reworking of pre-existing crust^3^,^4^,^6^,^9^,^18^,^19^.

## The Supercontinent Cycle

Continental crust is largely generated and destroyed in accretionary-type subduction margins^20^, and reworked within continental orogenic settings^2^. The secular variation in juvenile (I-type) versus reworked (S-type) magmatism is controlled by the predominance of one tectonic style over another. The principal geological driver of prevailing global tectonic style is the supercontinent cycle, which describes the episodic assembly of most or all of the continental masses on a timescale of 200–500 Myr^21^,^22^,^23^. The progression of a single cycle is recorded through the episodic dominance of subduction and supercontinent assembly leading to amalgamation (high degrees of crustal recycling resulting in evolved magma compositions), versus rifting and supercontinent dispersal leading to subduction and assembly of the next supercontinent (dominated by juvenile mantle additions). Thus, over the course of successive cycles a secular trend of juvenile versus evolved magmatism may be deciphered.

**Figure 1:**
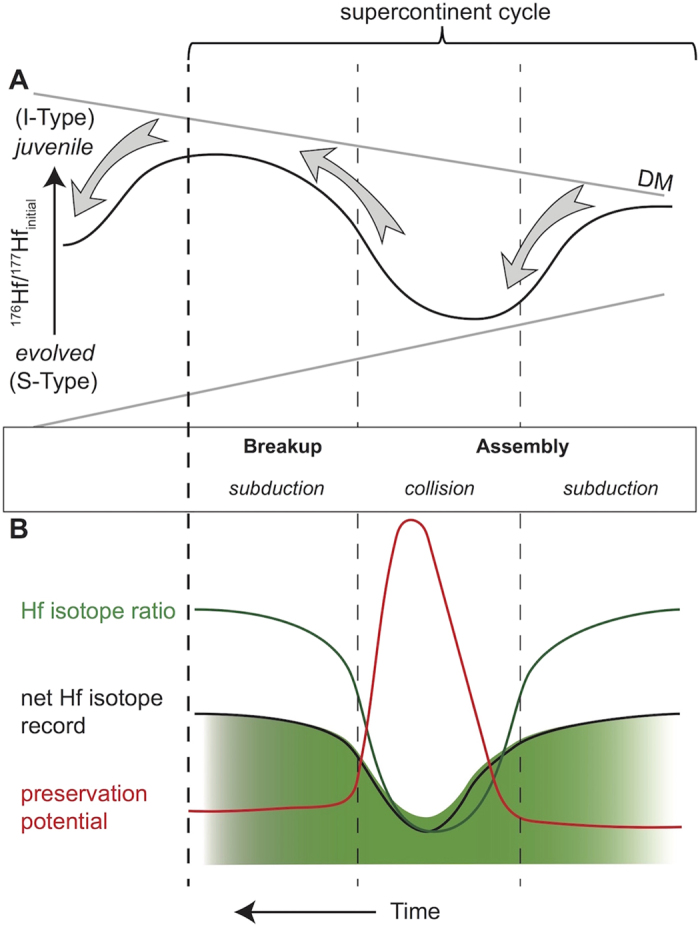
(**A**) Schematic showing the conceptual evolution of initial ^176^Hf/^177^Hf ratios with respect to the stages of supercontinent assembly and breakup. During initial assembly, subduction processes prevail, with the generation of predominantly juvenile magmatism leading to a more radiogenic Hf signature (analogous to I-type granites). As the supercontinent amalgamates, crustal thickening becomes increasingly dominant, with crustal reworking processes leading to more evolved magmatism during final assembly, driving global Hf towards more evolved ratios (S-types). Breakup and rifting processes leading to subduction give rise to more juvenile Hf signatures. (**B**) Diagram showing the proposed effect of preservation bias on the Hf signal during a supercontinent cycle. Preservation curve and volume of generated magma retained in the geological record *sensu* Hawkesworth *et al*. (2009). A lack of preservation will dilute global juvenile Hf signals towards a less radiogenic baseline, but will not affect the more evolved part of the record during continent collision. The net effect is to dampen but not remove the Hf isotope signal. The depth of shading reflects intensity of signal, based on number of analysis.

A pattern of changing magmatism accompanying a supercontinent cycle can also be proposed: (a) a prevalence of I-type arc magmatism accompanying widespread subduction as ocean basins close between accreting continents; (b) a dominance of crustal reworking processes and S-type magmatism accompanying collision during final supercontinent amalgamation; (c) rifting and MORB-type magmatism, leading to supercontinent breakup and renewed I-type magmatism related to subduction activity. A single supercontinent cycle thus drives a predictive pattern of Hf isotope ratios from juvenile through evolved, and back to juvenile (Fig. 1A).

## Results

### Calculation of the Juvenile Signal

**Figure 2:**
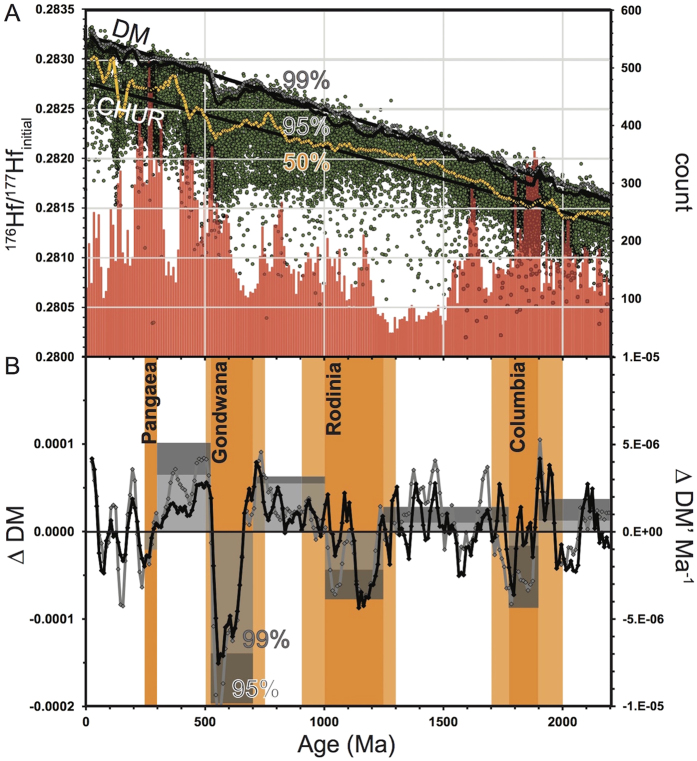
(**A**) Scatter plot of initial ^176^Hf/^177^Hf versus U-Pb magmatic age (Ma) for all datapoints from 0 to 2200 Ma. The 99th, 95% and median (50%) moving average timeseries are plotted. CHUR = Chondritic Uniform Reservoir; DM = Depleted Mantle. A frequency plot (red) of Hf analysis count per 10 Myr bin is also plotted (right scale). (**B**) ΔDM (left scale)–a de-trended difference between DM and the Hf signal per 10 Myr; black = ΔDM_95_; grey = ΔDM_99_. A histogram of the ΔDM’_99_ and ΔDM’_95_ signals, the time-integrated difference for the periods within and between the indicated supercontinent assemblies (right scale). Periods of supercontinent assembly shown, with timeline A in dark orange and timeline B in lighter orange.

38,303 zircon Hf analyses were extracted from the dataset of Roberts and Spencer^12^, representing the time interval 2200–0 Ma, and which encompasses the assembly of four major supercontinents: Pangaea, Gondwana, Rodinia and Columbia (Nuna). This dataset has global coverage, incorporates analyses of both magmatic and detrital zircon, and is perhaps the most complete dataset available, representing a near-continuous record of magmatism sampled from a variety of tectonic settings and localities (Fig. 2A). Data were compiled into 10 Myr bins, and for each bin, the following parameters were calculated and smoothed over 50 Myr: (a) the 99th percentile (99%; the most juvenile); (b) the 95% juvenile; and (c) the median (50%) (Fig. 2A). From these, a bin-level deviation from DM was calculated and de-trended for each timeseries (e.g., 99%-DM). The net result was three Hf isotope timeseries with respect to a DM baseline, here termed ΔDM_99_, ΔDM_95_ and ΔDM_50_. Positive ΔDM records an excursion to a more radiogenic Hf signal, reflecting greater mantle input into the magma; a negative ΔDM represents an excursion to a more evolved Hf signal (Fig. 2B).

### Correlation with Supercontinent Assembly

**Figure 3:**
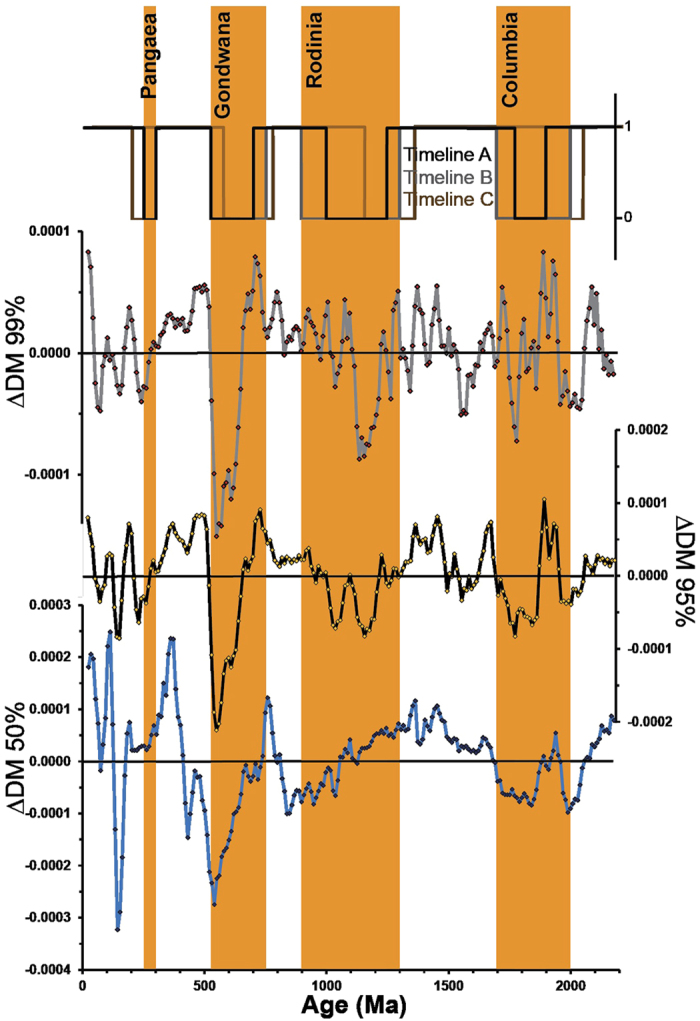
Correlation analysis. The supercontinent binary signal over time is compared to ΔDM_99_, ΔDM_95_ and ΔDM_50_. Supercontinent assembly timeline B is shown in orange.

We tested the correlation between each ΔDM timeseries, and intervals of supercontinent assembly and breakup using the point-biserial test^24^, which compares a continuous record (i.e. Hf), against a binary record (i.e. supercontinents) (Fig. 3). Estimation of ages of individual supercontinent cycles through Earth’s history have followed a number of independent lines of evidence: zircon U-Pb age data^25^; whole-rock Nd isotope data^14^; temporal distribution of high-grade metamorphic rocks^26^; extent of passive margins through time^27^; the Sr isotopic composition of seawater^14^,^27^; the incompatible trace element composition of magmas and oxygen isotope composition of zircons^13^. In order to test the sensitivity of our method to the timings of supercontinent assembly, we constructed and then calculated correlations between three different supercontinent assembly timelines: timelines A and B which reflect a tighter and looser assembly based on published data^4^,^10^,^13^,^14^,^27^,^28^,^29^,^30^, and timeline C, a shifted timeline that has an arbitrarily assigned offset of between 50 and 200 Myr per cycle, and which reflects a less geologically valid timeline (Table 1). For these assembly timelines, supercontinent assembly = 1, and breakup = 0.

For both timelines A and B, the point-biserial test (*n* = 218) indicates a statistically significant correlation at >99% confidence for all ΔDM timeseries (ΔDM_50_, ΔDM_95_, ΔDM_99_) between (a) radiogenic Hf excursions with breakup intervals and (b) evolved Hf excursions with assembly intervals. In statistical analysis, the p-value is a measure of correlation between two datasets, where (0 ≤ p ≤ 1) and a p-value < 0.1 suggests a >99% significance of correlation. The R-value is a measure of scatter of the correlation (−1 ≤ R ≤ 1), where 1 is a perfect positive correlation. Calculated R and p-values are given in Table 1. Our analysis indicates that choosing either timeline A or B, i.e. a tighter and looser interpretation of the same underlying supercontinent timeline does not significantly affect the correlation. Furthermore, the analysis also indicates that the ΔDM_95_ signal most accurately records timelines A and B through the smallest p-value. For timeline C, however, the test results suggest that shifting the supercontinent assembly timelines does significantly affect the correlation, since the p-values for all Hf timeseries show <95% confidence. These same variations are also observed with respect to a deviation normalized against CHUR as opposed to DM (Table 1), illustrating that the choice of baseline does not affect the correlation with the supercontinent cycle.

### Secular Trends

To identify trends through time, we interrogated the juvenile ΔDM signals within the framework of the supercontinent cycle as given by timeline A (Fig. 2B). For each interval of assembly and breakup, a time-integrated value of ΔDM_99_ and ΔDM_95_ was calculated (i.e. the area under the curve divided by the duration), here termed ΔDM’_99_ and ΔDM’_95_. These constructs permit comparison between successive cycles by measuring the gross deviation in the Hf signal during intervals of assembly and intervals of breakup.

Our results show that both ΔDM’_99_ and ΔDM’_95_ systematically record an excursion towards ever more evolved Hf signals during the intervals of final assembly of Columbia, Rodinia and Gondwana (histogram, Fig. 2B). The intervening intervals show excursions towards ever more juvenile Hf. This increase in signal amplitude with time occurs over the three successive supercontinent cycles until the onset of the final assembly of Pangaea at ca. 300 Ma. After this we observe: (a) a reduction in ΔDM’_99_ and ΔDM’_95_ at 300 Ma; and (b) a significant contraction in the ΔDM’_99_ and ΔDM’_95_ signals at ca. 250 Ma, on Pangaea breakup.

## Discussion

### Preservation Bias

Workers have proposed that population peaks in the U-Pb zircon record which correspond to intervals of supercontinent assembly are an artifact of preservation^9^,^31^,^32^. The preservation potential for early subduction-related (early assembly) and later extensional-related (breakup) magmatism is conceptually lower than for collision-related magmatism (i.e. late assembly). Roberts (2012)^4^ suggested a similar phenomenon affects the Hf record. However, here we propose that the global Hf record is less sensitive to selective preservation, and thus consideration of such a preservational phenomenon is arguably less crucial for interpretation of Hf isotope datasets.

**Figure 4:**
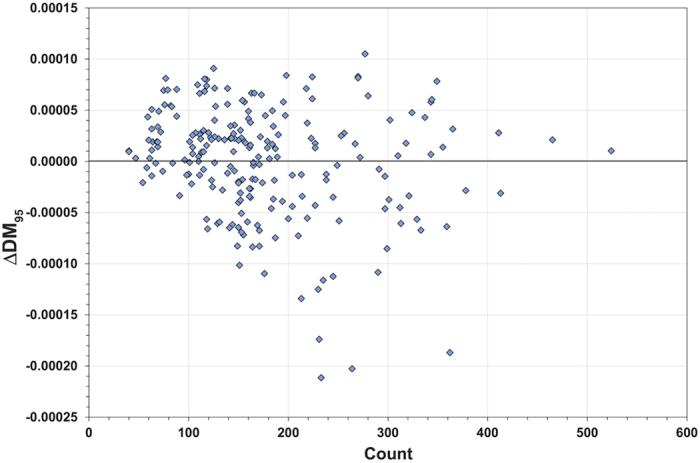
Plot of per bin count of Hf analysis versus ΔDM_95_ signal. No obvious correlation between the number of analysis and juvenile Hf signal is observed.

The zircon U-Pb record is based on the frequency of observations. Yet, selective preservation may erode the signal by reducing the number of analyses at a given age. In comparison, the Hf record is arguably less sensitive to the *count* of preserved analyses, and, depending on the statistical interrogation of the Hf dataset, may be more sensitive to their collective *excursion* within Hf isotope space. The pattern of low preservation potential affects those parts of a supercontinent cycle with a dominance of juvenile material. During these intervals, selective removal of some of the juvenile analyses through erosion may move the average of that group towards more evolved values. However due to the distribution of data, this process is unlikely to move the average to the type of values seen in those parts of the cycle with a dominance of evolved material (which also have the best preservation potential). Hence, the net result of any selective analysis removal from the dataset may be to dilute the juvenile part of the signal towards a global crustal balance at the median, but to still maintain a pattern of juvenile and evolved excursions through time (Fig. 1B). In addition, we find there is no correlation between the number of analyses per bin, and ΔDM_95_, which is consistent with a lack of significant preservation bias in the global Hf signal (Fig. 4).

### Assembly of the Supercontinents

**Figure 5:**
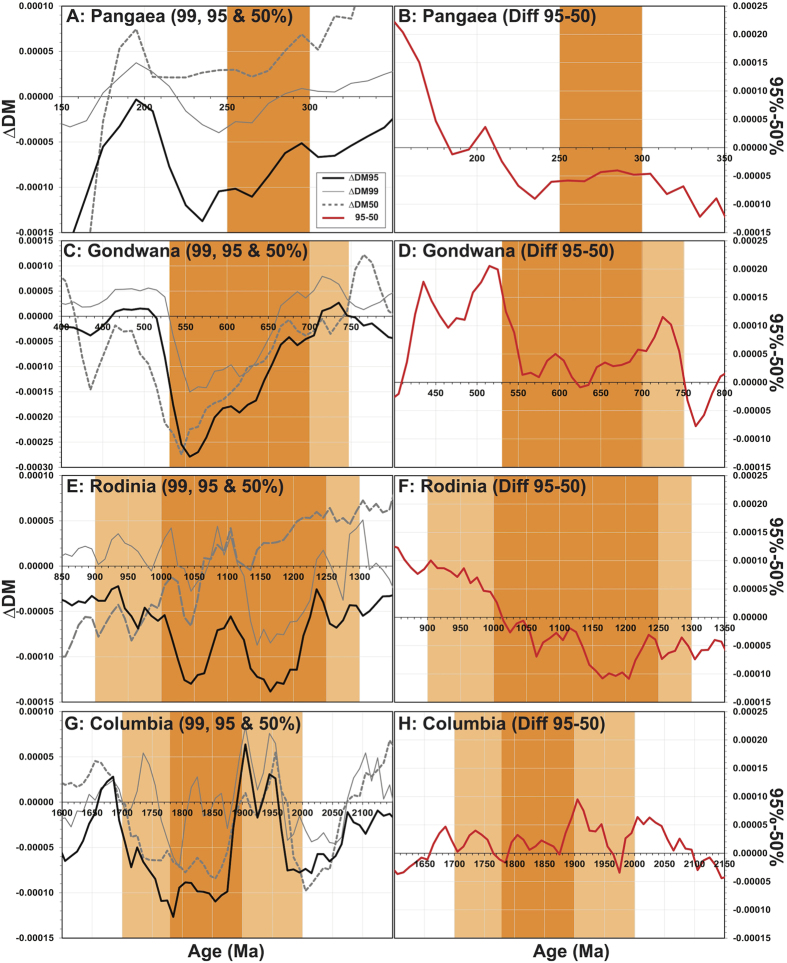
Detail of ΔDM_99,_ ΔDM_95_, and ΔDM_50_ (**A,C,E,G**), and of the difference between ΔDM_95_ and ΔDM_50_ (**B,D,F,H**), for the assembly times of the four supercontinents considered in this study. Periods of supercontinent assembly in dark orange are for timeline (**A**), and in light orange for timeline (**B**).

The sensitivity of the three ΔDM (50, 95 and 99) signals to the assembly of each of the supercontinents is shown in Fig. 5. Final assembly of Columbia between 1900–1780 Ma is arguably best recorded both by the juvenile 95% and by the median signals, since they both show excursions towards more explicitly evolved values with progression of assembly (Fig. 5G). Immediately prior to Columbia assembly, the Hf signals show a pronounced negative excursion between 2070–1970 Ma, suggestive of an extended period of continental reworking. This evolved excursion may support the existence of a Palaeo to Meso-Proterozoic supercontinent (e.g., Sclavia and/or Superia)^33^. The positive excursion that follows at 1900 Ma may reflect its breakup, as suggested by a peak in the abundance of passive margins^27^.

Inspection suggests differences in the median ΔDM_50_ and juvenile (ΔDM_95_ and ΔDM_99_) records with respect to the assembly of both Pangaea and Rodinia (Fig. 5A,C). Specifically, the median signal appears insensitive to the final assembly of Pangaea (300–250 Ma), in that it does not record a shift to evolved values. This observation has previously been interpreted as reflecting a balance between juvenile and evolved material^14^. However, our analysis suggests that final Pangaea assembly *is* recorded in both the juvenile Hf signals through the magnitude of their excursion towards more explicitly evolved values, especially through the 95% signal (Fig. 5A). Compared to Pangaea, Rodinia has a much more protracted interval of assembly (250 versus 50 Myr). The juvenile 95% Hf signal traces two pronounced excursions towards more evolved values, a pattern not observed in the median signal (Fig. 5E).

### The Case of Gondwana

We observe a trend in the gross deviation of Hf isotopic excursions with successive supercontinent cycles (i.e. that recorded by ΔDM’). This trend is suggestive of a signal increasingly biased towards more radiogenic and evolved values over time until and including Gondwana assembly, which of all the supercontinents exhibits the most evolved Hf signal (Fig. 2B). The observed differences between Rodinia, Gondwana and Pangaea assembly may be informed through understanding the relationship between the juvenile and median signals, as shown in Fig. 5(B,D,F,H). The arithmetic difference between the juvenile and median part of the signal at a point in time represents the spread of data points toward more evolved values, and is a measure of the heterogeneity of global magma volumes at any period in time. This is best described through the de-trended difference between the 95% and 50% Hf binned ratios (Fig. 5B,D,F,H).

During final Gondwana assembly, there is an accord between all three ΔDM signals (Fig. 5C). However, there is a positive spread between the juvenile ΔDM_95_ and median signal (Fig. 5D), in contrast to that seen for the assembly of Pangaea (Fig. 5B). Murphy and Nance^34^ suggested that observed differences in Nd model ages between Pangaea and Gondwana, reflected different modes of assembly: extroversion (Gondwana) versus introversion (Pangaea). Extroversion describes a supercontinent that reassembles through consumption and reworking of older oceanic material, and is consistent with the spread in Hf data for Gondwana which indicates the availability of more evolved material over global melt volumes.

**Figure 6:**
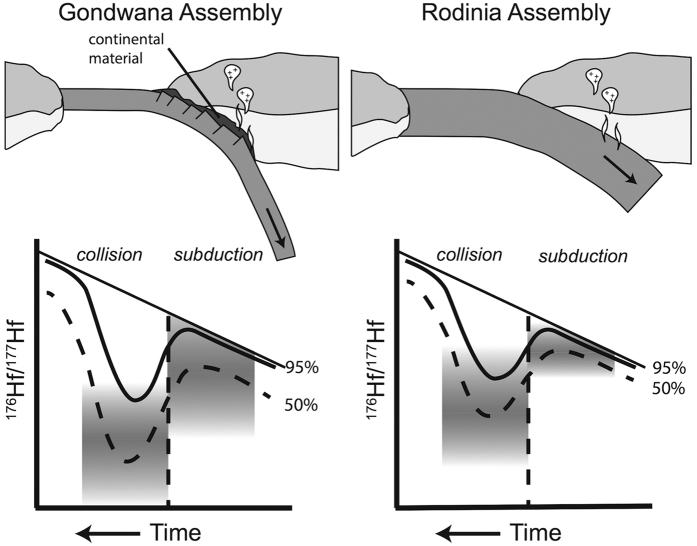
Schematic of possible differences in subduction style for Gondwana versus Rodinia. Thinner oceanic crust in the Phanerozoic leads to enhanced subduction-erosion and the involvement of existing crust in plate margin magmatism. This leads to a more evolved Hf signal during both early subduction, and later continent collision. The potential effect on the juvenile (95%) and median (50%) Hf signal is illustrated.

Gondwana is the supercontinent that exhibits the most evolved signal during assembly. Differences in the Hf record associated with Gondwana final assembly in comparison with that of Rodinia have been proposed as reflecting extensive reworking of Palaeoproterozoic and older crust^11^, specifically within the Pan-African orogeny^35^. While our data are consistent with this model, an alternate explanation for the significantly more evolved Gondwana assembly signal may be that it reflects a secular change in subduction style since the breakup of Rodinia. Oceanic crust formed since the Neoproterozoic is some 2–3 times thinner than that formed in the Mesoproterozoic and older^36^. Further, there is a suggestion that plates grew stepwise to a pentagonal configuration controlled by geoid highs^37^, widening the ocean basins. The combined effect of this process is colder, thinner, and less buoyant oceanic crust entering subduction zones during Gondwana assembly, leading to steeper, and colder, subduction, marked by the appearance of lawsonite eclogites in the rock record at this time^38^. This increase in subduction angle means enhanced subduction-erosion, ultimately leading to greater crustal recycling (Fig. 6). Furthermore, it is possible that steeper subduction may favour the process of cratonic lithospheric delamination, i.e. the loss of its cratonic root, such as that experienced by the Saharan metacraton during the Pan African Orogeny^39^. Such lithospheric delamination may then enhance reworking of the older cratonic crust, which in turn may accentuate the predominance of evolved Hf values as observed for final assembly of Gondwana. We thus suggest an increase in crustal recycling associated with steeper subduction may be observed in the pronounced Gondwana Hf excursion.

Pangaea would also experience this more modern-style steep subduction geometry, and the primacy of subduction-erosion as a crustal reworking process over new crust addition via arc magmatism in the Phaneorozic has been proposed^40^. However, the data show that the final assembly of Pangaea exhibits a relatively minor evolved Hf excursion compared to both Gondwana and Rodinia, and there are two possible contributions to this. Firstly, its relatively minor evolved Hf excursion may reflect its final assembly by dominantly introversion processes^34^. Secondly, the Central Asian Orogenic Belt (CAOB), the most major orogenic event during the Phanerozoic^41^, has been estimated to have an unusually high proportion of juvenile addition. Greater than 50% of the 500–100 Ma magmatism associated with the CAOB comprises a juvenile component of over 60%^42^, and this may contribute to an overall less evolved Hf signal for Pangaea assembly.

## Conclusions

Periodicities within the global zircon Hf signal correlate with high statistical confidence to the supercontinent cycle. Analysis of the assembly timeline of four supercontinents since 2200 Ma suggests that the Hf juvenile 95% signal may be most sensitive to collision and breakup processes, a feature which we interpret as a consequence of it being least affected by mixtures with crustal components. The total amplitude (e.g., the areas under the Hf curve) of the juvenile Hf signal during supercontinent cycles varies with time, reaching a maximum intensity immediately prior to the final assembly of Pangaea and markedly diminishing thereafter, and which may reflect a secular change in subduction style leading to enhanced subduction-erosion during Gondwana assembly. Enhanced subduction erosion would be predicted to result in enhanced crustal reworking, resulting in greater Hf isotopic heterogeneity over global magma volumes. The greater variability of Hf isotopes during Gondwana assembly, as recorded by the differences between the median and juvenile timeseries, may also be consistent with extroversion, with enhanced reworking of older oceanic material, rather than introversion as recorded by Pangaea. Broad scale secular excursions in Hf isotopes towards more juvenile or evolved signals can be used to infer globally prevailing tectonic style, much in the way that the I and S-type granite construct has been used to assist in inferences on geodynamic setting.

## Methods

### Calculation of Hf timeseries

Datapoints for the period 0–2200 Ma were extracted from the Hf dataset of Roberts and Spencer (2014)^12^. For each point, the initial ^176^Hf/^177^Hf ratio was calculated from the reported measured ^176^Hf/^177^Hf and ^176^Lu/^177^Hf ratios, and the determined U-Pb crystallization age, using the decay constant of Scherer *et al*. (2001)^43^. This recalculated dataset was put into 10 Myr buckets (e.g., 0–10 Ma; 10–20 Ma etc.) which reflect the crystallization age. Each bin represents a spread of initial Hf values between the most juvenile (highest initial Hf ratio) and most evolved (lowest). For this spread, the median (50%), the 95% and 99% were calculated, the latter two representing the most juvenile, i.e. highest initial Hf ratios. These values were then plotted against the mean age of the bucket (e.g., 5 Ma, 10 Ma etc.). A four-point running average for each timeseries was applied i.e. smoothing over a 50 Myr time period. Results from these steps are given in the Supplementary data (Table S1). For each timeseries the arithmetic difference from the Depleted Mantle (DM) reference line as defined per Griffin *et al*. (2000)^16^ was calculated (i.e. 99%-DM). These difference timeseries were then de-trended using linear regression techniques encapsulated in the de-trending algorithm in the statistical package Past 3^44^. This yielded ΔDM_99_, ΔDM_95_ and ΔDM_50_.

### Correlation Analysis

To calculate a correlation between a supercontinent timeline and the Hf signal we used the point-biserial test^24^. This allows comparison between a dichotomous and a continuous dataset. We constructed a timeseries dataset for each of the three timelines of supercontinent assembly using a binary 1 for breakup period and 0 for the assembly period, over the same time periods as for the Hf data (5–2195 Ma). The python function scipy.stats.pointbiserialr was used to calculate the correlation coefficients. The null hypothesis is that the two datasets are unrelated, and the p-value gives a value between 0 and 1 representing the probability that this null hypothesis is valid. Thus a p-value < 0.1 suggests a >99% significance that there is a correlation.

## Additional Information

**How to cite this article**: Gardiner, N. J. *et al*. The Juvenile Hafnium Isotope Signal as a Record of Supercontinent Cycles. *Sci. Rep.* **6**, 38503; doi: 10.1038/srep38503 (2016).

**Publisher’s note:** Springer Nature remains neutral with regard to jurisdictional claims in published maps and institutional affiliations.

## Supplementary Material

Supplementary Information

## Figures and Tables

**Table 1:** Supercontinent assembly timeline and correlation statistics.

Timeline	Pangaea	Gondwana	Rodinia	Columbia	ΔDM_99_	ΔDM_95_	ΔDM_50_	ΔCHUR_99_	ΔCHUR_95_	ΔCHUR_50_
Timeline A	300–250 Ma	700–530 Ma	1250–1000 Ma	1900–1780 Ma	R: 0.44777	R: 0.59392	R: 0.27807	R: 0.44571	R: 0.59241	R: 0.27715
					p: 3.80e-12	p: 3.57e-22	p: 3.12e-5	p: 4.90e-12	p: 4.83e-22	p: 3.32e-5
Timeline B	300–250 Ma	750–530 Ma	1300–900 Ma	2000–1700 Ma	R: 0.23542	R: 0.44602	R: 0.33425	R: 0.23276	R: 0.44402	R: 0.33308
					p: 4.56e-4	p: 4.71e-12	p: 4.35e-7	p: 5.31e-4	p: 6.02e-12	p: 4.80e-7
Timeline C	250–200 Ma	800–600 Ma	1350–1150 Ma	2050–1900 Ma	R: 0.09402	R: 0.04358	R: −0.0285	R: 0.09290	R: 0.04271	R: −0.0291
					p: 0.17170	p: 0.52211	p: 0.67555	p: 0.17170	p: 0.53046	p: 0.66967
