# Supplementary material for: The Juvenile Hafnium Isotope Signal as a Record of Supercontinent Cycles
Source: Sci Rep. 2016 Dec 7;6:38503. doi: 10.1038/srep38503 (PMC5141473; doi:10.1038/srep38503)
Supplement: Supplementary Information [file srep38503-s1.pdf]

The Juvenile Hafnium Isotope Signal as a Record of Supercontinent Cycles

Nicholas J. Gardiner[1,2*], Christopher L. Kirkland[1,2] and Martin J. Van Kranendonk[2,3]

1. Centre for Exploration Targeting – Curtin Node, Department of Applied Geology, Western Australian School of Mines, Curtin University, Perth, WA 6102, Australia

2. Australian Research Council Centre of Excellence for Core to Crust Fluid Systems, Australia.

3. School of Biological, Earth and Environmental Sciences, University of New South Wales Australia, Kensington, NSW 2052, Australia

** Corresponding author. E-mail address: nicholas.gardiner@curtin.edu.au*

Supplementary DataTable

Table S1: Datatable of smoothed 99%, 95%, 50% ^{176}Hf/^{177}Hf and count per 10 My bucket

Ma (bucket mean)	Hf CHUR(t)	Hf DM(t)	Count	Hf initial 99%	Hf initial 95%	Hf initial 50%
25	0.282755977	0.283232468	118	0.283298224	0.283234799	0.283013963
35	0.282749566	0.283225052	195	0.283278702	0.283205802	0.283032335
45	0.282743153	0.283217635	126	0.283229741	0.283180663	0.283016791
55	0.28273674	0.283210217	249	0.283168832	0.2831293	0.28293288
65	0.282730325	0.283202797	204	0.283141807	0.283112499	0.282879407
75	0.282723908	0.283195376	245	0.283131787	0.283083762	0.282782664
85	0.282717491	0.283187953	154	0.283161468	0.283093695	0.282825704
95	0.282711072	0.283180529	100	0.283162517	0.283091841	0.282870099
105	0.282704652	0.283173104	112	0.283170408	0.283124215	0.283001207
115	0.282698231	0.283165677	152	0.283144919	0.283120215	0.283022002
125	0.282691808	0.283158248	256	0.283142087	0.28311011	0.282837892
135	0.282685385	0.283150818	227	0.283124008	0.283032334	0.282630037
145	0.28267896	0.283143387	164	0.283102544	0.282984511	0.282430953
155	0.282672534	0.283135954	299	0.283088676	0.282975531	0.282458001
165	0.282666106	0.28312852	321	0.283088383	0.283019959	0.282556344
175	0.282659677	0.283121085	179	0.283111944	0.283065944	0.282706585
185	0.282653248	0.283113648	160	0.283121752	0.283080944	0.282780963

195	0.282646816	0.283106209	218	0.283130807	0.283103091	0.282795248
205	0.282640384	0.283098769	156	0.283113234	0.28308277	0.282735857
215	0.28263395	0.283091328	171	0.283090511	0.283014323	0.282728661
225	0.282627516	0.283083885	297	0.283056007	0.282963928	0.28272204
235	0.282621079	0.283076441	359	0.283033818	0.282939055	0.282720709
245	0.282614642	0.283068996	413	0.283017788	0.282964461	0.282716824
255	0.282608204	0.283061549	378	0.283022759	0.282959935	0.282710628
265	0.282601764	0.2830541	301	0.283014362	0.282943669	0.282696263
275	0.282595323	0.28304665	297	0.283028617	0.28295922	0.282696326
285	0.28258888	0.283039199	524	0.283032106	0.282976911	0.282711588
295	0.282582437	0.283031746	465	0.283030657	0.282980378	0.282723281
305	0.282575992	0.283024292	310	0.283020377	0.282957606	0.282699474
315	0.282569546	0.283016836	343	0.283012499	0.282951671	0.28272998
325	0.282563099	0.283009379	318	0.283014882	0.282955285	0.282720547
335	0.28255665	0.283001921	411	0.283010377	0.282958162	0.282777822
345	0.2825502	0.282994461	219	0.283010437	0.282960448	0.282748328
355	0.282543749	0.282986999	139	0.283009424	0.282971639	0.282820755
365	0.282537297	0.282979536	163	0.283003622	0.282975125	0.282843578
375	0.282530843	0.282972072	139	0.282992607	0.282972533	0.28283595
385	0.282524389	0.282964606	154	0.282978427	0.282953446	0.282733579
395	0.282517933	0.282957139	127	0.282977288	0.282940427	0.282673098
405	0.282511475	0.28294967	184	0.282966188	0.282928884	0.28265112
415	0.282505017	0.2829422	324	0.282963678	0.282919694	0.282586546
425	0.282498557	0.282934729	302	0.282946412	0.282905255	0.282487869
435	0.282492096	0.282927256	365	0.282939685	0.282889175	0.282415452
445	0.282485634	0.282919781	337	0.28293818	0.282893201	0.282454008
455	0.28247917	0.282912306	343	0.282941302	0.282900988	0.282488554
465	0.282472706	0.282904828	349	0.282952377	0.282913963	0.28252323
475	0.28246624	0.282897349	270	0.282945686	0.282911374	0.282504433
485	0.282459772	0.282889869	270	0.282939477	0.282902695	0.282499187
495	0.282453304	0.282882388	198	0.282928189	0.282897784	0.282446758
505	0.282446834	0.282874905	224	0.28292653	0.282889062	0.282420506
515	0.282440363	0.28286742	280	0.282915282	0.282863298	0.28236666
525	0.282433891	0.282859934	213	0.282894311	0.282779049	0.282289189
535	0.282427418	0.282852447	290	0.282809806	0.282676224	0.282261523
545	0.282420943	0.282844958	362	0.282742589	0.282590476	0.282213175
555	0.282414467	0.282837468	233	0.282683806	0.28255858	0.282256647
565	0.28240799	0.282829976	264	0.282686981	0.282560128	0.282255248
575	0.282401511	0.282822483	231	0.282677971	0.282581627	0.282285074
585	0.282395031	0.282814988	213	0.282703424	0.282614083	0.282289218
595	0.28238855	0.282807492	235	0.282699069	0.282624686	0.282288462
605	0.282382068	0.282799994	245	0.282701912	0.282621158	0.282297171
615	0.282375585	0.282792496	230	0.282671126	0.282601088	0.282307302
625	0.2823691	0.282784995	176	0.282673046	0.282609204	0.282333847
635	0.282362614	0.282777493	151	0.282685491	0.282610094	0.282331112
645	0.282356127	0.28276999	144	0.282708417	0.282642517	0.282332787
655	0.282349638	0.282762485	134	0.282732677	0.282668976	0.282351657
665	0.282343148	0.282754979	113	0.282776003	0.282698645	0.282388323
675	0.282336657	0.282747471	122	0.282782737	0.282705926	0.282394226
685	0.282330165	0.282739962	104	0.28278935	0.282682491	0.282365764
695	0.282323672	0.282732452	101	0.282769453	0.282686982	0.282349431
705	0.282317177	0.28272494	111	0.282777286	0.282686826	0.282352351
715	0.282310681	0.282717426	109	0.282798051	0.282727961	0.282369776
725	0.282304184	0.282709911	116	0.282785274	0.282726321	0.282332955

735	0.282297685	0.282702395	125	0.282767877	0.282729321	0.282350001
745	0.282291185	0.282694877	173	0.282730814	0.282696137	0.282364638
755	0.282284684	0.282687358	224	0.282709559	0.28268492	0.282441052
765	0.282278182	0.282679837	177	0.282695772	0.282661256	0.282463088
775	0.282271679	0.282672315	160	0.282696551	0.282658097	0.282440954
785	0.282265174	0.282664791	146	0.282696135	0.28263726	0.282382147
795	0.282258668	0.282657266	154	0.282702766	0.282615804	0.282331716
805	0.28225216	0.28264974	227	0.282703892	0.282604685	0.282312384
815	0.282245652	0.282642212	227	0.282687971	0.282592944	0.282320001
825	0.282239142	0.282634682	253	0.28266595	0.282597424	0.28226777
835	0.282232631	0.282627152	267	0.282630356	0.28258231	0.282236572
845	0.282226119	0.282619619	190	0.282633838	0.28258381	0.282186844
855	0.282219605	0.282612085	153	0.282630901	0.282573352	0.282180865
865	0.28221309	0.28260455	157	0.282620894	0.282561159	0.282190051
875	0.282206574	0.282597013	142	0.282622278	0.282558256	0.282202122
885	0.282200057	0.282589475	150	0.282617503	0.282548954	0.282204387
895	0.282193538	0.282581936	107	0.282607399	0.28254906	0.282196742
905	0.282187018	0.282574395	146	0.282582868	0.282536125	0.282169179
915	0.282180497	0.282566852	130	0.282581597	0.282528602	0.282175761
925	0.282173975	0.282559308	142	0.282595645	0.282533641	0.282181597
935	0.282167451	0.282551763	162	0.282594979	0.28252962	0.282184148
945	0.282160926	0.282544216	179	0.282577482	0.282497527	0.282162095
955	0.2821544	0.282536668	170	0.282566733	0.282481297	0.28213123
965	0.282147873	0.282529118	146	0.282553485	0.282460229	0.282136938
975	0.282141344	0.282521567	161	0.282534724	0.282475655	0.282143027
985	0.282134814	0.282514014	189	0.282517535	0.282459115	0.282150754
995	0.282128283	0.28250646	126	0.282529444	0.282446092	0.282140071
1005	0.28212175	0.282498904	110	0.282539171	0.282444865	0.282158566
1015	0.282115217	0.282491347	123	0.282543128	0.282414303	0.282160884
1025	0.282108682	0.282483788	204	0.282493911	0.282381362	0.282148946
1035	0.282102145	0.282476228	171	0.28248354	0.282350454	0.282102959
1045	0.282095608	0.282468667	155	0.282451415	0.282338803	0.282086953
1055	0.282089069	0.282461104	169	0.282455008	0.282340875	0.282109514
1065	0.282082529	0.28245354	129	0.282454088	0.282335234	0.28214805
1075	0.282075988	0.282445974	185	0.282467008	0.282351664	0.282140199
1085	0.282069445	0.282438406	111	0.282493951	0.282367645	0.282149267
1095	0.282062901	0.282430838	139	0.282454908	0.282362334	0.28213516
1105	0.282056356	0.282423268	96	0.282468304	0.282367824	0.282154068
1115	0.28204981	0.282415696	98	0.282431086	0.282345687	0.282111204
1125	0.282043262	0.282408123	124	0.28241014	0.28232669	0.282099807
1135	0.282036713	0.282400548	118	0.282353098	0.282287626	0.282087618
1145	0.282030163	0.282392972	119	0.28231906	0.282270856	0.282103081
1155	0.282023612	0.282385394	153	0.282329267	0.282260357	0.28210402
1165	0.282017059	0.282377815	149	0.282306808	0.282239417	0.282096998
1175	0.282010505	0.282370235	210	0.282309531	0.282242011	0.282091287
1185	0.28200395	0.282362653	187	0.28230128	0.282232686	0.282087074
1195	0.281997394	0.28235507	159	0.282307987	0.282240828	0.282089835
1205	0.281990836	0.282347485	131	0.282301521	0.282233133	0.282093846
1215	0.281984277	0.282339899	103	0.282304232	0.282263176	0.282091516
1225	0.281977717	0.282332311	84	0.282310127	0.282276031	0.282084232
1235	0.281971155	0.282324722	72	0.282347712	0.282299135	0.282084121
1245	0.281964592	0.282317131	69	0.282350701	0.282277229	0.28207101
1255	0.281958028	0.282309539	74	0.282328166	0.282245991	0.282074922
1265	0.281951463	0.282301945	63	0.282302975	0.282234021	0.282053025

1275	0.281944896	0.28229435	58	0.282273585	0.282234519	0.282050302
1285	0.281938328	0.282286753	40	0.282339155	0.282243594	0.282036375
1295	0.281931759	0.282279155	40	0.282337975	0.282235527	0.282044082
1305	0.281925189	0.282271556	67	0.282340132	0.282216762	0.282049339
1315	0.281918617	0.282263955	47	0.282277957	0.282214224	0.282031348
1325	0.281912044	0.282256352	63	0.282277803	0.282214765	0.282032022
1335	0.28190547	0.282248749	64	0.282263016	0.282215346	0.28201574
1345	0.281898895	0.282241143	68	0.282245412	0.282208008	0.282011869
1355	0.281892318	0.282233536	60	0.282221373	0.282202246	0.282031346
1365	0.28188574	0.282225928	82	0.282251205	0.282228429	0.282042817
1375	0.281879161	0.282218318	88	0.282276424	0.282237019	0.282045766
1385	0.28187258	0.282210707	83	0.282285104	0.2822127	0.281960128
1395	0.281865998	0.282203094	59	0.282259024	0.282195209	0.281949362
1405	0.281859415	0.28219548	70	0.282248434	0.282193332	0.281951321
1415	0.281852831	0.282187864	63	0.282215797	0.282187829	0.281981759
1425	0.281846245	0.282180247	63	0.282191512	0.282161294	0.281963125
1435	0.281839658	0.282172629	69	0.28218627	0.28215582	0.281947683
1445	0.28183307	0.282165009	76	0.282209887	0.282170007	0.28196698
1455	0.281826481	0.282157387	75	0.282215444	0.282176579	0.281976152
1465	0.28181989	0.282149764	77	0.282226894	0.28218096	0.281974352
1475	0.281813298	0.28214214	79	0.282191545	0.282162292	0.281953416
1485	0.281806705	0.282134514	88	0.282162962	0.28212899	0.281933875
1495	0.28180011	0.282126886	69	0.282155742	0.28209643	0.281924203
1505	0.281793514	0.282119257	54	0.282142336	0.282049291	0.281888379
1515	0.281786917	0.282111627	61	0.282155289	0.282065749	0.28187617
1525	0.281780319	0.282103995	101	0.282125562	0.282054185	0.281863992
1535	0.281773719	0.282096362	115	0.282126992	0.282077909	0.28186412
1545	0.281767118	0.282088727	145	0.282114192	0.282049859	0.281855299
1555	0.281760516	0.282081091	165	0.282092298	0.282028874	0.281827213
1565	0.281753913	0.282073453	91	0.282047663	0.281992	0.281829498
1575	0.281747308	0.282065814	164	0.282043616	0.28200065	0.281820163
1585	0.281740702	0.282058173	150	0.282033788	0.281990764	0.281816077
1595	0.281734095	0.282050531	165	0.282057625	0.282002082	0.28180319
1605	0.281727486	0.282042888	167	0.282051285	0.281977935	0.281791823
1615	0.281720876	0.282035242	238	0.2820341	0.281975593	0.281787151
1625	0.281714265	0.282027596	291	0.282044211	0.281973057	0.281775761
1635	0.281707653	0.282019948	272	0.282036152	0.281977233	0.281772715
1645	0.281701039	0.282012298	223	0.282047849	0.281988385	0.281775863
1655	0.281694424	0.282004647	185	0.282034125	0.281992603	0.281784072
1665	0.281687808	0.281996995	154	0.282040407	0.282010516	0.281775077
1675	0.28168119	0.281989341	116	0.282036397	0.282011896	0.281758093
1685	0.281674572	0.281981685	118	0.282034161	0.282009706	0.28174501
1695	0.281667952	0.281974028	104	0.282016414	0.28195401	0.281713588
1705	0.28166133	0.28196637	110	0.281984059	0.28192117	0.28170187
1715	0.281654708	0.28195871	162	0.281980883	0.281886902	0.281659142
1725	0.281648084	0.281951049	142	0.281992313	0.281901056	0.281654171
1735	0.281641459	0.281943386	150	0.282027032	0.281877539	0.281622936
1745	0.281634832	0.281935722	152	0.282006942	0.281860197	0.281613307
1755	0.281628205	0.281928056	150	0.281976603	0.281843382	0.281606119
1765	0.281621576	0.281920389	141	0.281930424	0.281811307	0.281599126
1775	0.281614945	0.28191272	150	0.281902114	0.281804166	0.281602992
1785	0.281608314	0.28190505	171	0.281875222	0.281778411	0.281583659
1795	0.281601681	0.281897378	153	0.281856188	0.281803019	0.281572879
1805	0.281595047	0.281889705	312	0.281901525	0.281801157	0.281558515

1815	0.281588411	0.28188203	183	0.281929905	0.281792757	0.281559348
1825	0.281581775	0.281874354	219	0.281934053	0.281775845	0.28156086
1835	0.281575137	0.281866676	200	0.281883438	0.281767725	0.281547168
1845	0.281568498	0.281858997	251	0.2818788	0.28175818	0.281527817
1855	0.281561857	0.281851317	333	0.281888388	0.281741623	0.281517344
1865	0.281555215	0.281843634	313	0.281886359	0.281740706	0.281522234
1875	0.281548572	0.281835951	329	0.28184006	0.281737273	0.281533522
1885	0.281541928	0.281828266	355	0.281866526	0.281800255	0.281559782
1895	0.281535282	0.281820579	344	0.281903633	0.281839363	0.281572427
1905	0.281528635	0.281812891	277	0.281930134	0.281876526	0.281577488
1915	0.281521987	0.281805202	111	0.281884951	0.281830345	0.281551824
1925	0.281515338	0.281797511	126	0.281845594	0.281780437	0.281537855
1935	0.281508687	0.281789818	197	0.281857011	0.281793723	0.281553122
1945	0.281502035	0.281782124	126	0.281893088	0.281812941	0.281560238
1955	0.281495381	0.281774429	166	0.28187469	0.281800548	0.281587634
1965	0.281488727	0.281766732	115	0.2818121	0.281735778	0.28153819
1975	0.281482071	0.281759034	162	0.281752922	0.281683608	0.281518355
1985	0.281475414	0.281751334	161	0.281752357	0.281677109	0.281451638
1995	0.281468755	0.281743632	152	0.281765079	0.281665935	0.281431944
2005	0.281462095	0.28173593	214	0.281741549	0.281662273	0.281400647
2015	0.281455434	0.281728225	194	0.281721618	0.28164982	0.281401341
2025	0.281448772	0.281720519	238	0.281717147	0.281663159	0.281403634
2035	0.281442108	0.281712812	186	0.281716716	0.28165539	0.281405374
2045	0.281435443	0.281705103	161	0.281698508	0.281639787	0.28139659
2055	0.281428777	0.281697393	174	0.281689838	0.2816379	0.281418975
2065	0.28142211	0.281689681	115	0.281689548	0.281643239	0.28144448
2075	0.281415441	0.281681968	145	0.281724677	0.281670886	0.281451797
2085	0.281408771	0.281674253	162	0.281739938	0.281652023	0.281451136
2095	0.2814021	0.281666537	187	0.281742759	0.281641117	0.281442713
2105	0.281395427	0.281658819	181	0.281752718	0.281623816	0.28146204
2115	0.281388753	0.2816511	118	0.281714226	0.281629214	0.281451183
2125	0.281382078	0.281643379	120	0.281732441	0.281634122	0.281451433
2135	0.281375401	0.281635657	112	0.281679512	0.281620554	0.281452285
2145	0.281368723	0.281627933	172	0.28168765	0.281615017	0.281470176
2155	0.281362044	0.281620208	184	0.281648322	0.281600275	0.28145299
2165	0.281355364	0.281612481	143	0.281652037	0.281598579	0.28144899
2175	0.281348682	0.281604753	104	0.281628567	0.28158219	0.281435787
2185	0.281341999	0.281597023	136	0.281632032	0.28158205	0.28146122
2195	0.281335315	0.281589292	123	0.2816141	0.281574778	0.281446336
